# The elevation of plasma concentrations of apoB-48-containing lipoproteins in familial hypercholesterolemia is independent of PCSK9 levels

**DOI:** 10.1186/s12944-017-0502-x

**Published:** 2017-06-15

**Authors:** Jean-Philippe Drouin-Chartier, Jean-Charles Hogue, André J. Tremblay, Jean Bergeron, Benoît Lamarche, Patrick Couture

**Affiliations:** 1 Institute of Nutrition and Functional Foods (INAF), 2440, Hochelaga Blvd, Pavillon des Services, Quebec City, G1V 0A6 Canada; 2 Lipid Research Centre, CHU de Québec-Université Laval, Quebec City, Canada

**Keywords:** Familial hypercholesterolemia, Apolipoprotein B-48, PCSK9, Atherosclerosis

## Abstract

**Background:**

Previous studies have reported high plasma concentrations of both intestinal apolipoprotein (apo) B-48-containing lipoproteins and PCSK9 in subjects with familial hypercholesterolemia (FH). However, the extent to which LDL receptor deficiency and PCSK9 levels influence plasma apoB-48 concentrations in humans remains to be fully characterized. The objective of the study was to assess the independent association between FH, PCSK9 concentrations and plasma apoB-48 levels in a large cohort of genetically defined FH heterozygotes (HeFH) and homozygotes (HoFH).

**Methods:**

A total of 118 HeFH, 6 HoFH, and 117 controls were included in the study. Plasma PCSK9 and apoB-48 concentrations were measured in the fasting state.

**Results:**

Plasma PCSK9 and apoB-48 levels were higher in FH subjects compared with controls (PCSK9: HoFH: 642.6 ± 246.9 vs. HeFH: 324.9 ± 119.8 vs. controls: 194.5 ± 65.9 ng/mL, *P* < 0.0001; apoB-48: HoFH: 14.71 ± 4.36 vs. HeFH: 6.55 ± 4.24 vs. controls: 3.03 ± 2.07 μg/mL; *P* < 0.0001). There were no correlations between apoB-48 and PCSK9 plasma levels in both controls (ρ = 0.06, *P* = 0.5) and HeFH subjects (ρ = 0.07, *P* = 0.4). Multiple linear regression analysis showed that the FH status was the only independent factor associated with apoB-48 levels, contributing to 28.7% of the variance (*P* < 0.0001).

**Conclusions:**

These data indicate that the elevation in plasma apoB-48 levels associated with FH is independent of PCSK9 levels.

**Trial registration:**

NCT02225340.

## Background

Familial hypercholesterolemia (FH) is an autosomal codominant single-gene disorder caused by mutations in the LDL receptor (LDLR) gene, its ligand apolipoprotein (apo) B, or proprotein convertase subtilisin/kexin type 9 (PCSK9) that disrupt normal clearance of LDLs [1, 2]. Phenotypic features of the disease’s heterozygous form (HeFH) are 2- to 3-fold raise in plasma LDL-cholesterol (C) concentrations, tendinous xanthomatosis and premature atherosclerotic coronary heart disease (CHD), usually occurring between the age of 35 and 55 years if untreated. Homozygous (HoFH) or compound heterozygous patients have plasma LDL-C concentrations that are 6- to 8-folds higher than normal and usually manifest a CHD event before the age of 20 years [3]. In the Province of Quebec (Canada), nine mutations are responsible for 90% of the FH cases in the French Canadian population, defined on the basis of clinical and biochemical criteria [4].

High concentrations of LDL particles are undisputedly associated with accelerated atherosclerosis [5]. Intestinal apoB-48-containing lipoproteins are also involved in the development of atherosclerosis and subsequent CHD [6–8]. Chylomicrons are too large to enter the subendothelial space, but once hydrolyzed by the lipoprotein lipase, chylomicron remnants of less than 700 Ǻ are small enough to enter into the intima and to participate to atherogenesis [9]. Chylomicron remnants have been shown to impair normal endothelial function [10], to be chemically modified and to accumulate in the subendothelial space the same way than apoB-100-containing lipoproteins [7, 11]. Chylomicron remnants are removed from the circulation by the LDLR-related protein (LRP) [12]. There is accumulating evidence reporting that FH patients have increased fasting apoB-48 levels [13–17], but data on the underlying mechanisms in cause are mixed.

Proprotein convertase subtilisin/kexin type 9 (PCSK9) plays a major role in lipoprotein clearance by promoting intracellular lysosomal degradation of the LDLR, the VLDL receptor (VLDLR) and the LRP [18, 19]. Concomitantly, PCSK9 stimulates chylomicron secretion in intestinal cells [20–22]. Studies from our group and others reported higher levels of PCSK9 in FH subjects [23, 24]. However, the extent to which LDL receptor deficiency and PCSK9 levels influence plasma apoB-48 concentrations in humans remains to be fully characterized.

The objective of the present study was to determine the independent association between FH, plasma PCSK9 levels and apoB-48 levels in a large cohort of HeFH, HoFH and healthy control subjects. We hypothesized that the LDLR deficiency in FH patients is associated with elevated plasma levels of apoB-48 and that PCSK9 levels are positively correlated with fasting apoB-48 levels in FH and controls.

## Methods

The study was approved by the Laval University Medical Center ethical review committee and informed consent was obtained from each patient. This trial was registered at http://clinicaltrials.gov as NCT02225340.

### HeFH subjects

A total of 118 HeFH (55 men and 63 women) were recruited for the present study. All HeFH patients were previously screened for the nine French Canadian mutations in the LDLR gene using genomic DNA at the Lipid Research Center of the CHU de Québec-Université Laval. The deletion >15 kb at the 5′ end of the gene [25] and the 5 kb deletion in the exons 2 and 3 [26] were analyzed by Southern blotting [27]. The seven point mutations were analyzed by restriction enzyme fragment analysis [4, 28].

To be eligible for the present study, HeFH subjects had to be at least 18 years of age. HeFH subjects also had to be homozygote for apoE3. Genotyping of apoE was done by PCR-amplification of a 244 bp fragment of the exon 4 of the apoE gene with oligonucleotides F4 and F6 and digestion of PCR fragments with the restriction enzyme HhaI [29]. HeFH subjects were ineligible if they had no confirmed LDLR gene mutations; a history of cardiovascular disease; were pregnant or nursing; had acute liver disease, hepatic dysfunction, or persistent elevations of serum transaminases; had plasma triglyceride levels >4.5 mmol/L; had a secondary hyperlipidemia due to any cause; had a recent history of alcohol or drug abuse; had diabetes mellitus; had a history of cancer; or had hormonal treatment.

Of the 118 HeFH subjects selected, 66 had the deletion >15 kb at the 5′ end of the gene [25], 37 had the W66G mutation in exon 3 [30], 8 had the Y468X mutation in exon 10 [28], 3 had the C646Y mutation in exon 14 [26], 1 had the C347R mutation in exon 8 [4], 1 had the C152W mutation in exon 4 [4], 1 had the R329X mutation in exon 7 [4], and 1 had the 5 kb deletion in the exons 2 and 3 [26].

All HeFH had to withdraw their lipid-lowering medications for at least 6 weeks before blood sample collection.

### HoFH subjects

A total of 6 HoFH (two men and four women) were recruited for the present study. Three were homozygotes for the >15 kb deletion, one was homozygote for the C660X Lebanese non-sense mutation [26, 31], one was homozygote for the W66G missense mutation, and one was a compound heterozygote for the >15 kb deletion and the W66G mutation. HoFH were under treatment with statin and, in some cases, ezetimibe and were all undergoing lipid apheresis treatment every 2 weeks.

### Control subjects

Control subjects (*n* = 117) were selected among the 2056 participants of the Quebec Health Survey, which comprised non-institutionalized men and women, excluding aboriginal populations, selected from health insurance files [32]. The Quebec Health Survey was designed to obtain relevant information on the prevalence and distribution of cardiovascular disease risk factors in the Quebec population, as previously described [32]. All control subjects were healthy, free of lipid-lowering medication, and were also homozygote for apoE3. Control subjects were selected to match FH subjects for gender distribution, age and BMI.

### Plasma lipids and lipoproteins

Blood samples were collected after a 12-h fast. They were collected immediately before a lipid apheresis treatment in HoFH patients. Blood samples were collected in tubes containing Na_2_EDTA [33]. Samples were immediately centrifuged at 4 °C for 10 min at 3000 rpm to obtain plasma and were stored at 4 °C until processed. Cholesterol and TG levels were determined in plasma and lipoprotein fractions by enzymatic methods (Randox Co., Crumlin, UK) using a RA-1000 analyzer (Bayer Corporation Inc., Tarrytown, NY), as previously described [34]. Plasma VLDL (d < 1.006 g/mL) were isolated by preparative ultracentrifugation and the HDL fraction was obtained after precipitation of LDL in the infranatant (d > 1.006 g/mL) using heparin and MnCl_2_. The cholesterol and TG content of the infranatant fraction were measured before and after the precipitation step.

### Quantification of total plasma apoB, apoB-48 and PCSK9

Total plasma apoB concentrations were determined by a commercial enzyme-linked immunosorbent assay (ELISA) kit using immuno-purified polyclonal antibodies (Alerchek Inc., Portland, Maine, USA). Plasma apoB-48 levels were determined using a commercial ELISA kit using immuno-purified monoclonal antibodies (Shibayagi Co., Shibukawa, Gunma Prefecture, Japan) and no cross-reactivity with apoB-100 (<0.001%) has been reported [35]. Plasma PCSK9 concentrations were measured using a commercial ELISA kit (Circulex, CycLex, Nagano, Japan).

### Power calculation

Power calculation was conducted on the expected association between plasma apoB-48 and PCSK9 levels as primary outcome. We previously reported a significant association between total apoB concentrations and PCSK9 levels (*r* = 0.31) in HeFH subjects [23]. Our power analysis indicated that the probability to detect a true change in plasma apoB-48 of 0.31 units per unit change in PCSK9 levels with 118 HeFH subjects at a two-sided 0.05 significance level was 91%. This calculation was conducted with a conservative approach by considering standard deviations of 40% for PCSK9 and apoB-48 levels [36].

### Statistical analyses

Plasma lipid and PCSK9 concentrations of the controls, HeFH and HoFH were compared using ANOVA models with the Tukey’s adjustment for multiple comparisons. Multiple linear regression model was used to assess independent associations between multiple variables and apoB-48 concentrations. Normality of the models was evaluated by the distribution of the scaled residual values. Spearman’s rank correlation test was used to evaluate the association between plasma apoB-48 and PCSK9 levels in controls and HeFH. All analyses were performed using JMP Pro software (v12.2.0, SAS Institute, Cary, NC). Statistical significance was considered at *P* < 0.05.

## Results

Table 1 presents the demographic and anthropometric characteristics of the 118 HeFH, 6 HoFH and 117 control subjects. The three groups were matched for age, gender distribution and BMI. The percentage of past or current smokers was higher in controls than in FH subjects (74.4 vs. 47.5%; *P* < 0.0001).

**Table 1:** Demographic and genotypic characteristics of subjects according to their status

	Controls	HeFH	HoFH	*P*
Subjects (*n*)	117	118	6	
Age (y)	38.7 ± 16.8	37.7 ± 11.2	27.0 ± 10.7	0.1
Sex				0.1
Men (%)	68 (58.1)	55 (46.6)	2 (33.3)	
Women (%)	49 (41.9)	63 (53.4)	4 (66.7)	
BMI (kg/m^2^)	25.2 ± 4.5	25.4 ± 4.2	24.6 ± 2.7	0.9
LDLR mutations				
Del >15 kb (%)	-	66 (55.9)	-	
W66G (%)	-	37 (31.4)	-	
Y468X (%)	-	8 (6.8)	-	
C646Y (%)	-	3 (2.5)	-	
C347R (%)	-	1 (0.8)	-	
C152W (%)	-	1 (0.8)	-	
R329X (%)	-	1 (0.8)	-	
Del 5 kb (%)	-	1 (0.8)	-	
Double Del >15 kb (%)	-	-	3 (50.0)	
Double W66G (%)	-	-	1 (16.7)	
Double C660X (%)	-	-	1 (16.7)	
Del 15 kb + W66G	-	-	1 (16.7)	
Smoking				<0.0001
Ever (%)	87 (74.4)	56 (47.5)	0	
Never (%)	30 (25.6)	62 (52.5)	6 (100.0)	

Data are listed as mean ± SD. Percentages are indicated in parentheses. All control and HeFH subjects were apoE3 homozygotes. *HeFH* heterozygotes for familial hypercholesterolemia, *HoFH* homozygotes for familial hypercholesterolemia, *BMI* body mass index, *LDLR* LDL receptor, *Del* deletion

**Fig. 1:**
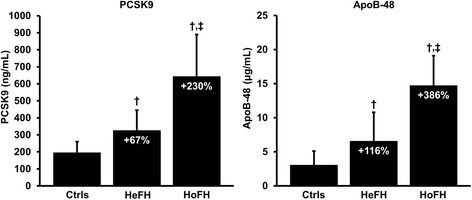
Relative increase in concentrations of plasma PCSK9 and plasma apoB-48 in HeFH and HoFH subjects vs. control subjects. Values are presented as mean ± standard deviation. Ctrls: controls; HeFH: heterozygotes for familial hypercholesterolemia; HoFH: homozygotes for familial hypercholesterolemia; apo: apolipoprotein; PCSK9: proprotein convetase subtilisin/kexin type 9. †*P* < 0.05 vs. controls. ‡*P* < 0.05 vs. HeFH

Table 2 shows the biochemical characteristics of each group. FH subjects had significantly higher concentrations of total-C, LDL-C, apoB, apoB-48 and PCSK9 and significantly lower HDL-C concentrations compared with controls. Fasting TG concentrations were similar between the three groups. HoFH subjects had higher concentrations of total-C, LDL-C, apoB, apoB-48 and PCSK9 than HeFH subjects. Figure 1 shows that PCSK9 levels were +67% higher in HeFH and +230% higher in HoFH subjects compared with controls (*P* < 0.05, for both). Similarly, apoB-48 levels were +116% and +386% higher in HeFH and HoFH subjects, respectively, compared with controls (*P* < 0.05, for both). Compared with HeFH subjects, PCSK9 and apoB-48 levels were +98% and +125% higher in HoFH subjects (*P* < 0.05, for both).

**Table 2:** Fasting biochemical characteristics of subjects according to their status

	Controls	HeFH	HoFH	*P*
Total-C (mmol/L)	5.21 ± 0.92	8.55 ± 1.64^*^	10.54 ± 2.41^*,**^	<0.0001
LDL-C (mmol/L)	3.22 ± 0.85	6.89 ± 1.51^*^	8.88 ± 2.58^*,**^	<0.0001
HDL-C (mmol/L)	1.30 ± 0.31	1.03 ± 0.26^*^	1.02 ± 0.34^*^	<0.0001
Plasma triglycerides (mmol/L)	1.53 ± 0.71	1.52 ± 0.79	1.42 ± 0.56	0.9
Plasma apoB (mg/dL)	118.8 ± 50.9	207.1 ± 138.3^*^	601.3 ± 203.7^*,**^	<0.0001
Plasma apoB-48 (μg/mL)	3.03 ± 2.07	6.55 ± 4.24^*^	14.71 ± 4.36^*,**^	<0.0001
Plasma PCSK9 (ng/mL)	194.5 ± 65.9	324.9 ± 119.8^*^	642.6 ± 246.9^*,**^	<0.0001

Data are listed as mean ± SD. All control and HeFH subjects were apoE3 homozygotes. *HeFH* heterozygotes for familial hypercholesterolemia, *HoFH* homozygotes for familial hypercholesterolemia, *C* cholesterol; apo: apolipoprotein, *PCSK9* proprotein convertase subtilisin/kexin type 9

^*^ *p* < 0.05 vs. controls

^**^ *p* < 0.05 vs. HeFH

**Fig. 2:**
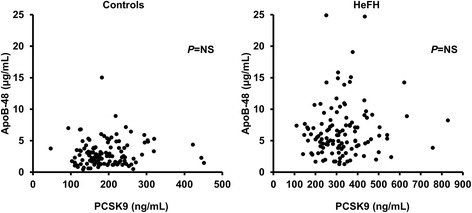
Correlations between plasma apoB-48 and PCSK9 concentrations in control subjects and HeFH subjects. HeFH: heterozygotes for familial hypercholesterolemia; apo: apolipoprotein; PCSK9: proprotein convertase subtilisin/kexin type 9. NS: non-significant

The association between plasma apoB-48 and PCSK9 concentrations was first assessed in controls and HeFH separately. As presented in Fig. 2, fasting plasma apoB-48 and PCSK9 levels were not correlated in neither of the two groups. There was also no association between apoB-48 and PCSK9 concentrations among HeFH subjects with the del15kb mutation and among those with the W66G mutation (data not shown). The correlation between apoB-48 and PCSK9 concentrations was not assessed in HoFH subjects and in HeFH subjects with C646Y, C347R, C152W, R329X, Y468X or del5kb mutation because of the limited number of subjects (*n* < 10).

**Fig. 3:**
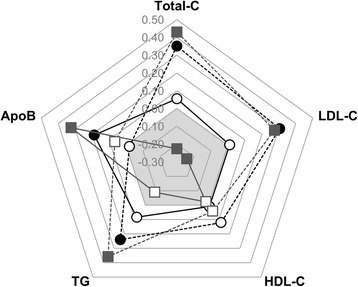
Radar plot presenting the associations among fasting apolipoprotein B-48 levels, PCSK9 concentrations and various plasma lipids in control subjects and HeFH subjects. *Radar lines* represent Spearman’s correlation coefficient. *Continuous lines* represent apoB-48 concentrations. *Dashed lines* represent PCSK9 concentrations. *Circles* represent HeFH subjects. *Squares* represent control subjects. *Filled marks* identify significant association (*P* < 0.05), and *white-filled marks* represent non-significant association. Apo: apolipoprotein; C: cholesterol; HeFH: heterozygotes for familial hypercholesterolemia; TG: triglyceride

In HeFH subjects, fasting plasma apoB-48 levels were positively associated with plasma apoB concentrations (*r* = 0.18, *P* = 0.04), but no association was measured between fasting apoB-48 and TG levels (*r* = 0.08, *P* = 0.4) (Fig. 3). In control subjects, apoB-48 concentrations were also positively associated with plasma levels of apoB (*r* = 0.32, *P* = 0.002). In both HeFH and control subjects, PCSK9 levels were positively associated with concentrations of total-C, LDL-C and TGs.

Using a multiple linear regression analysis, the independent association between plasma apoB-48 levels and variables physiologically susceptible to modulate them, namely the FH status, BMI, LDL-C and PCSK9 levels, age and sex, was assessed. The FH status (control vs. HeFH vs. HoFH) was the only significant multivariate correlate of fasting apoB-48 levels, accounting for 28.7% of its variance (Table 3). Differences in apoB-48 concentrations between controls, HeFH and HoFH remained statistically significant in this multiple linear regression model. Finally, among HeFH subjects only, the LDLR mutation was not a significant covariate of apoB-48 levels (data not shown).

**Table 3:** Multivariate regression analysis showing independent contribution of FH status, BMI, PCSK9, gender and age on the variance of fasting plasma apolipoprotein B-48 concentrations

Independent variables	Partial (R^2^ X 100)	*P*
FH status	28.7	<0.0001
LDL-C levels	1.7	0.1
BMI	0.8	0.3
Sex	0.7	0.3
PCSK9 levels	0.4	0.4
Age	0.0	0.8
Total	32.3	<0.0001

FH status refers to controls vs. HeFH vs. HoFH. *FH* familial hypercholesterolemia, *BMI* body mass index, *PCSK9* proprotein convetase subtilisin/kexin type 9

## Discussion

In the present study, the independent association between LDLR deficiency, PCSK9 levels and the variability of plasma apoB-48 concentrations was assessed in a large cohort of genetically defined FH patients and controls. HeFH and HoFH had respectively two- and five-fold higher plasma apoB-48 levels than controls. The FH status was the only independent factor associated with plasma apoB-48 levels, explaining 28.7% of its variance. No association was found between plasma PCSK9 and apoB-48 levels. These data suggest that the increase in fasting apoB-48 levels associated with FH is independent of PCSK9 levels.

Intestinal apoB-48-containing chylomicron remnants contribute to atherogenesis and constitute a significant risk factor for CHD [6, 8]. Large chylomicrons are unable to enter the subendothelial space, but chylomicrons remnants are small enough to enter into the intima and to participate in atherosclerosis [6, 9]. The over-accumulation of small dense cholesterol-rich chylomicron remnants, caused by an impaired clearance or an increased secretion, is associated with increased arterial exposure, permeability, retention and subsequent accumulation within the vessel wall [7]. In agreement with previous studies [13–15, 37], the present results demonstrated that LDLR deficiency is associated with high fasting levels of plasma apoB-48-containing lipoproteins. Thus, in addition to elevated LDL-C concentrations, it is likely that the high plasma apoB-48 levels contribute significantly to CHD risk in FH subjects.

PCSK9 undisputedly plays a major role in lipoprotein clearance by regulating the concentration of cell receptors, namely the LDLR, VLDLR and LRP [18, 19]. In this context, the role of PCSK9 in chylomicron metabolism was evaluated in the past years and some investigators observed an association between PCSK9 levels and chylomicron clearance. Le May et al. [22] reported a lower postprandial response in PCSK9 knockout mice compared to wildtype mice following a fat load, which suggested that PCSK9 reduces chylomicron clearance. Similarly, in 17 obese humans, Chan et al. [38] recently reported an inverse association between fasting PCSK9 levels and the fractional catabolic rate of apoB-48-containing triglyceride-rich lipoproteins.

Recent evidence also suggested that PCSK9 promotes chylomicron secretion. Levy et al. [21] and Rashid et al. [20] both reported increased cellular and secreted apoB-48 in CaCo-2/15 cells treated with PCSK9. PCSK9 induced an upregulation in mRNA expression and protein levels of both apoB-48 and microsomal triglyceride transfer protein. It was suggested that the intracellular cholesterol depletion induced by PCSK9-mediated LDLR degradation stimulated cholesterol uptake from the intestinal lumen and activated chylomicron synthesis and secretion pathways [20, 21]. Although apoB-48 levels were higher in FH subjects compared with controls, data from the present study do not relate this over-accumulation to PCSK9. In this context, the high concentrations of fasting apoB-48 associated with FH is likely to be independent of PCSK9. A possible explanation could be related to the activity of plasma PCSK9. In healthy subjects, it was observed that approximately 40% of circulating PCSK9 is associated to LDLs and that this association inhibits the PCSK9-mediated lipoprotein-receptor degradation [39]. It was also observed that some hepatic furins cleave and inactivate plasma PCSK9 [40]. Overall, plasma PCSK9 concentrations do not directly reflect PCSK9 biological activity [41]. That could explain the absence of correlation between PCSK9 and apoB-48 concentrations observed in the present study.

It is also possible that the impact of PCSK9 on apoB-48 secretion occurs mainly in the postprandial state. Reyes-Soffer et al. recently reported that alirocumab, a monoclonal antibody to PCSK9, had no impact on postprandial apoB-48 response in healthy subjects [42]. While these observations are concordant with results of the present study, an extensive assessment of the association between PCSK9 and apoB-48 in the postprandial state remains required in FH patients.

Discrepancies exist regarding the mechanisms involved in the chylomicron remnant accumulation in FH subjects. Some authors reported that higher levels of apoB-48 levels in FH result from a clearance defect [13, 15, 17, 43]. On the other hand, a number of investigators reported no delayed clearance in apoB-48-containing lipoproteins associated with LDLR deficiency. Rubinsztein et al. [44], using vitamin A-labelled chylomicrons in HoFH subjects, reported no change in chylomicrons catabolism and no significant correlation was observed between LDLR activity of cultured fibroblasts collected from these patients and their retinyl palmitate response. Furthermore, Watts et al. [15] and Eriksson et al. [37] reported that the capacity of chylomicron and chylomicron remnant clearance was not affected by variation in LDLR activity. Twisk et al. [45] showed, in cultured hepatocytes of LDLR^−/−^ mice, an increase in the production rate of apoB-100 and apoB-48 and reported that this increased secretion of apoB resulted from a greater proportion of newly synthesized apoB escaping degradation. Their findings suggest that the LDLR mediates degradation of apoB-100 and apoB-48 before secretion and also mediates reuptake and degradation of newly secreted apoB. Because of the absence of the putative LDLR binding domain on apoB-48, it is unlikely that the LDLR modulates the proportion of apoB-48 escaping presecretory degradation. However, it is likely that a proportion of apoB-48 escapes the reuptake mechanism in the presence of LDLR defect, via interaction between the LDLR and apoE, leading to an increase in the production rate [45]. This observation was corroborated to some extent in vivo using apoB labelling with stable isotope and multicompartmental modelling by Tremblay et al. [16]. The production rate of intestinal lipoproteins was enhanced and catabolism was unaltered in FH subjects, which resulted in a 1.8-fold higher postprandial apoB-48 levels [16]. One can speculate that the impact of LDLR deficiency on intracellular cholesterol depletion is more potent than the effect of the PCSK9-mediated LDLR degradation. In this context, compensatory mechanisms measured in PCSK9-treated intestinal cells promoting chylomicron synthesis and secretion would be similar to those induced by FH.

An interesting hypothesis has been proposed by Sniderman et al. [46] regarding the “apoB paradigm”, which states that the rate at which LDL particles are produced is an important determinant of their concentration in plasma as their clearance rate. The “apoB paradigm” also states that secretion of cholesterol within VLDL particles is an important mechanism to maintain cholesterol homeostasis within the hepatocyte. Recycling of cholesterol could occur in the hepatocyte to prevent accumulation of cholesterol within the liver and could explain the increased secretion of cholesterol-rich apoB particles in patients with FH [46]. Recycling of cholesterol without expansion of the regulatory pool would also explain why cholesterol synthesis continues in the liver of patients with homozygous FH. The course of chylomicrons could be similar in the enterocyte and an increase in apoB-48 production rate may be one of the factors explaining the accumulation in the fasting state found in FH patients. This hypothesis is supported by data from the kinetic study by Tremblay et al. [16] described above.

This study encompasses several strengths and limitations. The large number of subjects genetically defined for FH and homozygotes for apoE3 is one of the major strengths. Also, the assessment of apoB-48 by ELISA without cross-reactivity with apoB-100 (<0.001%) is another strength. Most of the previous studies on chylomicron metabolism in FH subjects used ultracentrifugation to isolate and quantify chylomicrons, which prevent the assessment of small chylomicron remnants found in denser fractions. Data on circulating apoB-48 are presented on the basis of only a single blood sample, however, and no data on the diet in the days preceding the blood sample were available, which may have induced variation in plasma apoB-48 levels. Moreover, the measurement of plasma PCSK9 by ELISA did not allowed to quantified the relative proportion of furin-cleaved, LDL-associated or free PCSK9 as well as the overall biological activity of circulating PCSK9. Finally, the absence of postprandial measurements of apoB-48 may limit the interpretation of the present results.

## Conclusion

In summary, these findings demonstrated that the over-accumulation of fasting apoB-48-containing lipoproteins in genetically defined FH patients is independent of variations in plasma PCSK9 levels. Kinetic studies and clinical trials on the impact of PCSK9 inhibitors on fasting and postprandial apoB-48 concentrations are required to corroborate observations of the present study.

## Abbreviations

Apo: Apolipoprotein
BMI: Body mass index
C: Cholesterol
CHD: Coronary heart disease
Ctrl: Control
ELISA: Enzyme-linked immunosorbent assay kit
FH: Familial hypercholesterolemia
HeFH: Heterozygous familial hypercholesterolemia
HoFH: Homozygous familial hypercholesterolemia
LDLR: LDL-receptor
LRP: LDL-receptor-related protein
PCR: Polymerase chain reaction
PCSK9: Proprotein convertase subtilisin/kexin type 9
TG: Triglyceride
VLDLR: VLDL receptor
